# How Reliable Is Breast Volume Assessment When the Patient Is Lying Flat?—Volumetric Assessment of Breast Volume Using a Vectra H2 Handheld Device in Different Positions

**DOI:** 10.3390/jcm13030709

**Published:** 2024-01-25

**Authors:** Aljosa Macek, Sebastian Leitsch, Konstantin Christoph Koban, Julius Michael Mayer, Rafael Loucas, Thomas Holzbach

**Affiliations:** 1 Department of Hand and Plastic Surgery, Thurgau Hospital Group, 8500 Frauenfeld, Switzerlandthomas.holzbach@stgag.ch (T.H.); 2 Division of Hand, Plastic and Aesthetic Surgery, University Hospital LMU Munich, 80339 Munich, Germany; 3 Department of Plastic and Hand Surgery, Inselspital, University Hospital Bern, 3010 Bern, Switzerland

**Keywords:** breast volume, breast operation, Vectra, 3-D volumetric assessment, breast volumetry, Vectra H2 handheld device

## Abstract

(1) **Background**: Three-dimensional (3D) volumetric assessment is receiving increased recognition in breast surgery. It is commonly used for preoperative planning and postoperative control with the patient standing in an upright position. Recently, intraoperative use was evaluated with patients in the supine position. The aim of this prospective study was to evaluate the volumetric changes in 3D surface imaging depending on the patient’s position. (2) **Methods**: 3D volumetric analysis was performed using a Vectra-H2 device with patients in standing, sitting, and supine positions. A total of 100 complete datasets of female breasts were included in the study. The measured volumes of each evaluated breast (*n* = 200) were compared between the three positions. (3) **Results**: The mean difference between the 3D volumetric assessments of the sitting and standing positions per breast was 7.15 cc and, thus, statistically insignificant (*p* = 0.28). However, the difference between supine and standing positions, at 120.31 cc, was significant (*p* < 0.01). (4) **Conclusions**: The 3D volumetric assessment of breasts in the supine position did not statistically correlate with the validated assessment of breast volume in the standing position while breast volume in the sitting position is reliable and correlates with the assessment of a standing patient. We conclude that intraoperative volumetric assessment should be performed with patients in an upright sitting position.

## 1. Introduction

Breast operations, such as reduction mammoplasty, augmentation, mastopexy, lipofilling, and others, represent a very large proportion of procedures in plastic surgery. Besides the height of the nipple-areolar complex and scar positions, breast volume is one of the key aspects in determining breast symmetry [1,2,3].

The accurate and reliable assessment of breast volume is essential for achieving satisfactory surgical outcomes [4]. Various methods have been developed to measure breast volume, including mammography, magnetic resonance imaging (MRI), and 3D volumetric analysis [5,6,7]. In the last two decades, several authors relied on 3D surface imaging [8,9,10,11]. Initially, the 3D surface imaging devices were large, immobile, and costly. Moreover, it was impossible to use these devices in the operating theatre to intraoperatively evaluate symmetry. Recently, advances in technology have introduced available mobile handheld devices, such as the Vectra H2, for 3D volumetric assessment. These devices offer a convenient and effective method for measuring anthropometric measurements [12], and enable the surgeons to perform volume assessments intraoperatively.

Breast volume assessment plays a crucial role in various aspects of breast surgery, including preoperative planning, the intraoperative assessment of symmetry, and the postoperative evaluation of the results [10]. Usually, the surface imaging-based volume measurements are performed on a standing patient, both pre- and postoperatively [9,10]. Due to obvious reasons, intraoperative volume measurement is not feasible with the patient in a standing position. At our department, we routinely bring our patients to an upright position intraoperatively to evaluate the symmetry. However, some authors have suggested performing intraoperative measurements with the patient in a supine position [13].

Understanding the differences and correlations between measurements in different positions is crucial for accurate interpretation and decision-making during breast surgery. The objective of this prospective study was to compare the volumetric assessments of breast volume in both sitting and supine positions with the standard validated standing position [14,15]. The aim was to determine whether the intraoperative measurements correlated with those taken in the standing position.

## 2. Materials and Methods

This is a prospective study of the 3D volumetric assessment outcomes in standing, sitting, and supine positions of patients undergoing breast surgery, including reduction mammaplasty, breast augmentation, mastopexy, and breast lipofilling. The procedures were all performed in a standardized manner by four senior board-certified plastic surgeons in our department.

Between January 2022 and February 2023, a standardized 3D volumetric assessment in the above-mentioned three positions was routinely performed at our outpatient clinic on all the patients undergoing breast surgery, both preoperatively and postoperatively, to evaluate breast volume.

We evaluated the same breast at the same time in three different positions. Some of the datasets were acquired before an operation, some were acquired after an operation, and very few datasets were acquired without any operation planned or previously performed.

We used the VECTRA^®^ H2 3D imaging system (Canfield Scientific^®^, Parsippany, NJ, USA). It is a commercially available handheld (weight = 400 g) 3D camera with in-built infrared sensors for depth sensing with a capture volume of 700 mm (H) × 410 mm (W) × 400 mm (D) and ranging lights for easier capture distancing. The device comes with software for 3D picture analyses (VECTRA Analysis Module).

The technical requirement for the VECTRA volumetric 3D surface imaging system software (Canfield Scientific, Parsippany, NJ, USA) is a Windows computer with a processor of a minimum of 2.75 GHz dual-core (although quad-core is recommended). Additionally, the system memory (random-access memory) has to be at least 16 GB. For the software installation, at least 1 GB of available disk space is required, and a disk space of 1 TB is recommended for data storage. The graphics card should be at a minimum of 1 GB (however, 2 GB is recommended). A dedicated nVidia or AMD card is required. Regarding the computer driver, the latest available version, OpenGL 3.0 or Open GL 1 Gbps, is required. Furthermore, the 2 USB ports (USB 2.0) and a display with a 1920 × 1080 resolution are required (however, a display with a 1920 × 1200 resolution is recommended). Regarding the operating system, a Windows 10 Professional 64-bit or Windows 11 Professional 64-bit is recommended, as Windows Home Edition and Windows 7 are not supported. Furthermore, an ethernet connection for networking is needed; Wi-Fi is not supported.

**Figure 1:**
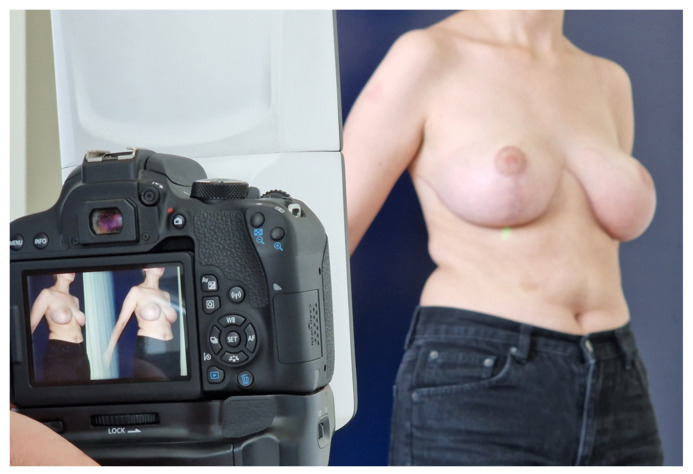
Taking the lateral image of a patient positioned correctly in a standing position (standing upright, back leaning to a wall, with arms slightly abducted and palms placed on the wall).

**Figure 2:**
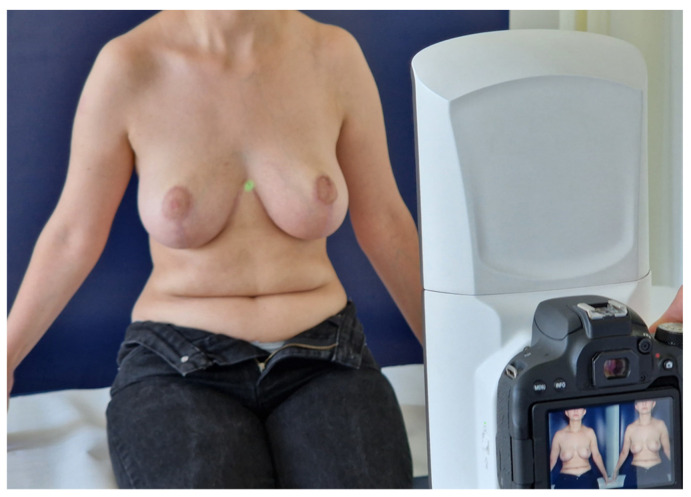
Taking the frontal image of a patient positioned correctly in a sitting position (sitting with the upper body upright at an angle of 90°, and as with the standing position, arms slightly abducted and hands positioned symmetrically).

Initially, the patient was positioned standing upright with their arms slightly abducted to ensure an unobstructed view of the lateral part of the breast. The patient’s head was oriented in a neutral position, allowing the proper exposure of body landmarks, such as the clavicles and sternal notch (Figure 1). Then, two lateral (45° from the midline, with the target point in the center of each inframammary fold, caudally cranially orientated) and one frontal (with the target point right beneath the caudal border of sternum images were taken in all three positions (Figure 2). We took the images from the standardized distance (defined usingthe VECTRA^®^ H2 Device using two intersecting lasers) and angles (−45°/0°/45°) to capture the whole surface area of both breasts as well as all the relevant body landmarks.

After that, the images were uploaded to a computer, and a 3D analysis was performed using the VECTRA analysis module program (Canfield Scientific, Parsippany, NJ, USA). Breast volume assessments were calculated for each breast in all three positions and then compared between the different positions.

Our institutional breast database documents 321 consecutive breast patients between January 2020 and February 2023. Of these, 151 patients were assessed in the outpatient clinic using 3D volumetric measurements in three different positions. We excluded all the patients with Regnault breast ptosis grade two or more since the 3D reconstruction with these seemed to produce more artifacts.

The ethical commission of eastern Switzerland (EKOS) conducted an assessment of the study and concluded that it did not raise any ethical concerns.

### Statistical Analyses

The volume assessments of each breast in the sitting and supine positions were compared to the measured volumes in the validated standing position using the *t*-test for paired samples. Furthermore, the differences in the volumes between the right and left breasts in the sitting and supine positions were compared to the measured differences in the standing position using the same test. All statistical analyses were performed using IBM^®^ SPSS^®^ statistical software (Version 29.0; IBM^®^, Chicago, IL, USA). A difference of *p* < 0.05 was considered statistically significant.

## 3. Results

A total of 100 complete datasets from 87 female patients with a mean age of 46.5 years (range: 17–80) and a mean BMI of 24.71 kg/m^2^ (range: 18.07–36.00) were included in the study. The 3D volumetric assessment using the Vectra H2 handheld device was performed in three positions: sitting, supine, and standing. The measured volumes of each evaluated breast (*n* = 200) were compared between the three positions. A total of 184 breasts were evaluated postoperatively, and 16 breasts were evaluated preoperatively (Table 1).

**Figure 3:**
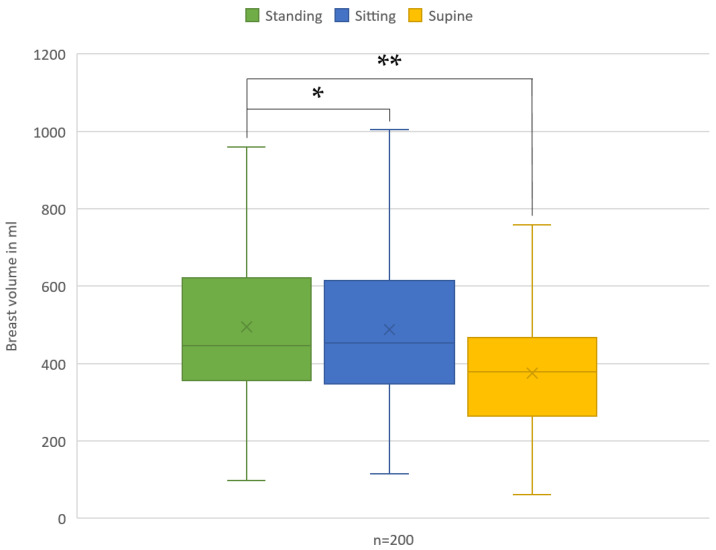
Graphical representation of the mean breast volumes for each position. * *p* > 0.05, ** *p* < 0.01.

The mean breast volume that was assessed while standing was 495.44 ± 204.46 cc, whereas in the sitting position, it measured as 488.28 ± 208.14 cc, indicating no statistically significant difference (*p* = 0.729). Conversely, in the supine position, the mean breast volume measured 375.13 ± 148.03 cc, and thus demonstrated a statistically significant difference from the standing position (*p* < 0.001) (Figure 3).

The mean absolute difference of the measured breast volumes per breast between the standing and sitting positions was 7.15 ± 93.67 cc (*p* = 0.281). This suggests that the 3D volumetric assessment of patients in the sitting position is reliable and correlates well with the validated assessment performed on a standing patient (Table 2).

**Figure 4:**
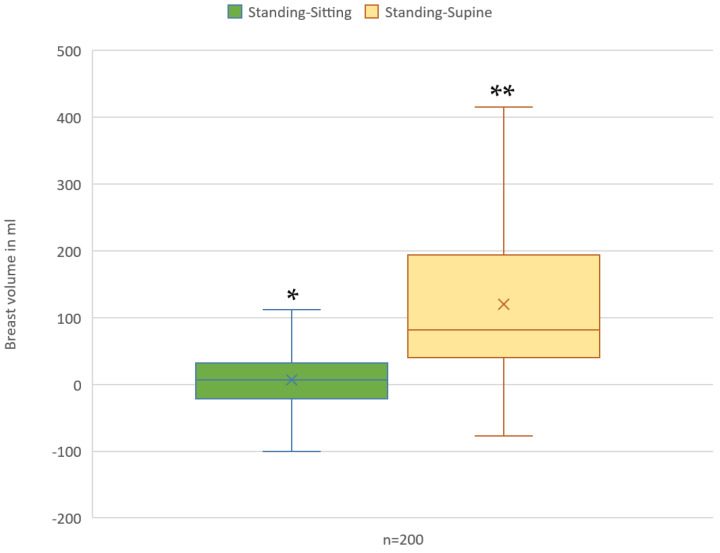
Graphical representation of the median differences of the measured breast volumes per breast between the different positions. * *p* > 0.05, ** *p* < 0.01.

In contrast, the mean difference in the measured volumes between the standing and supine positions per breast was statistically significant at 120.31 ± 113.06 cc (*p* < 0.001). These results suggest that the breast volumes measured in the supine position do not statistically correlate with the volumes obtained in the standing position (Figure 4).

**Figure 5:**
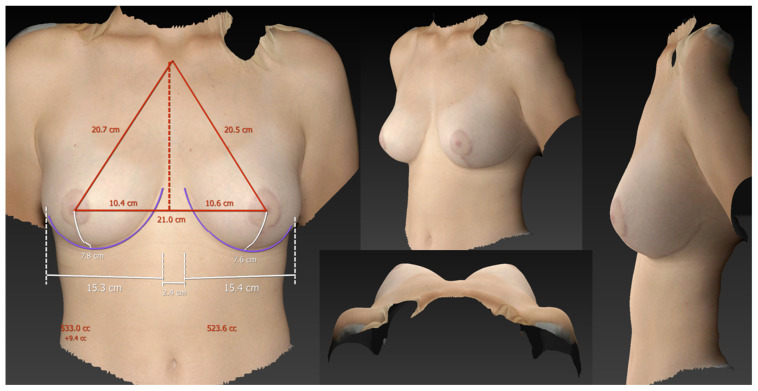
3D volumetric assessment of a patient six months after a reduction mammaplasty (patient 1) in the standing position. The measured breast volumes were 533 cc on the right side and 523 cc on the left side, showing a very symmetrical result.

**Figure 6:**
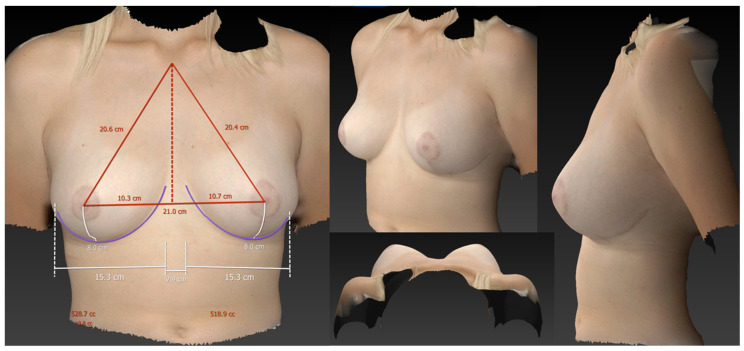
3D volumetric assessment of patient 1 in the sitting position. The measured breast volumes were 529 cc on the right side and 519 cc on the left side, thus correlating very closely with the volumetric assessment in the standing position. Additionally, note the very similar reconstructed shape of the breast compared to the standing position.

**Figure 7:**
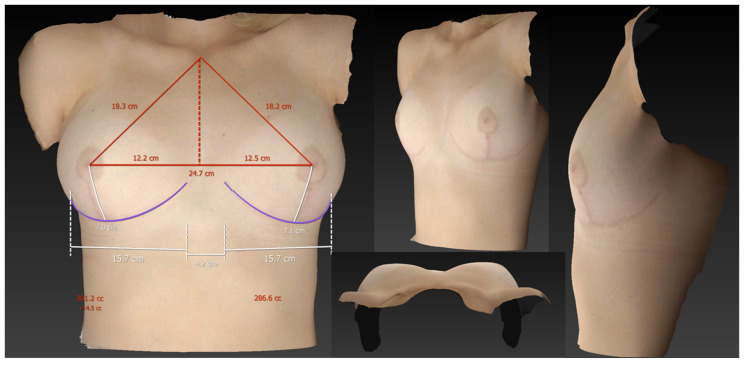
3D volumetric assessment of patient 1 in the supine position. The measured breast volumes were 301 cc on the right side and 287 cc on the left side, thus indicating a significant deviation from the volumes in the standing and sitting positions. Additionally, it is worth noting the striking disparity in the shape of the breast in the supine position compared to the other two positions.

The measured volume differences between the right and left breasts were found to be 11.34 ± 77.25 cc in the standing position, 2.06 ± 119.37 cc in the sitting position, and 0.85 ± 47.19 cc in the supine position. There were no statistically significant differences observed either between the standing and sitting positions (*p* = 0.16) or between the standing and supine positions (*p* = 0.14) (Figure 5, Figure 6 and Figure 7).

## 4. Discussion

In this study, we showed that the 3D breast volume measurement in a sitting position correlates with the measurement in the standard standing position. This indicates that the intraoperative measurement of a patient in an upright position is reliable and accurate.

Traditionally, surgeons had to rely on 2D anthropometric measurements and subjective optic estimations to evaluate breast volume and symmetry intraoperatively [14,16,17]. However, these methods have been shown to be subjective and prone to errors [15,18].

In recent years, there has been a notable shift in the approach to evaluating breast volume and symmetry. 3D surface imaging has emerged as a valuable tool for objectively quantifying the volume, shape, contour, and symmetry of the breast region [17,19,20,21]. The Vectra imaging system that we use in our department has already been validated for measuring breast volume and symmetry by O’Connor et al. [14].

Normally, 3D measurements are performed in a standing position, which can be performed for preoperative planning and postoperatively to evaluate the results. However, to evaluate the symmetry intraoperatively, one has to rely on the measurements in a sitting or supine position [22]. We have already conducted and published a study on the intraoperative volumetric assessment of breast volume using the Vectra device, where the assessments were performed in an upright sitting position [23]. Our primary objective in this study was to evaluate the accuracy and correlation of volumetric assessments in different patient positions.

Yang et al. proposed conducting intraoperative volume assessments while the patient is in the supine position [13]. However, we have observed that the breast shape and volume cannot be properly evaluated in the supine position. Even before the introduction of 3D volumetric measurements, we had always brought the patients to an upright position mid-operation for the evaluation of symmetry and shape. The outcome of this study, which demonstrates a statistically significant difference between the measured volumes in the standing and supine positions, confirms this observation. This is, to our knowledge, the first study comparing the measured volumes in different positions.

When comparing the median differences in the measured breast volumes between the right and left breasts, we observed no statistically significant differences across all three positions. This might imply that 3D volumetric measurement can be employed for the intraoperative assessment of breast symmetry in the supine position. However, the measured absolute volume in the supine position was significantly lower than in the upright position. Consequently, we suspect that relevant volumetric differences (detectable in the upright position) could go unnoticed when exclusively analyzing the breast in the supine position, leading to the detection of a low-percentage volumetric difference in a falsely low absolute volume. Moreover, it is important to note that the vast majority of the evaluated breasts (184 out of 200, or 92%) were postoperative results. This might introduce a bias to the statistics, as postoperatively, we mostly have very symmetric results, rendering the difference between the right and left breasts small.

### Limitations

Nevertheless, we acknowledge that there are several limitations that need to be discussed. Firstly, we did not exclusively analyze unoperated breasts. One could argue that after an operation, the breast tissue behaves differently in changing positions. To overcome this limitation, we did not measure breasts in the first three months after an operation to exclude alterations such as swelling or hematoma. Our data supports our presumption that it is justifiable to volumetrically analyze a breast after this period of time. Volume shifts in different positions are comparable to those of unoperated breasts.

Secondly, we recognize that the measured volumes do not represent the actual breast volumes. This is due to the inability to evaluate the thoracic wall using surface imaging. As a result, volume assessments rely on computer-generated models of the posterior breast borders, rendering them estimations rather than exact measurements. Nonetheless, it is worth noting that multiple authors have demonstrated a strong linear correlation between 3D surface scans and various methods of measuring breast volume, such as MRI-measured breast volume [24,25], mastectomy surgical specimens [25], and even water displacement [26].

Thirdly, there appears to be a limitation in acquiring correct 3D images when analyzing breasts that are ptotic. Here, it is not always possible to acquire full surface images of the inframammary fold or the lateral breast. For this reason, patients with ptotic breasts (Regnault grade 2 or more) were excluded from this study. However, our primary goal in utilizing this technique is to evaluate breast volumes and symmetry intraoperatively. Here, we usually do not see breast ptosis.

Furthermore, it is worth emphasizing that proper patient positioning is extremely important for reliable volume assessment. In the standing position, the patient should be standing upright, back leaning to a wall, with their arms slightly abducted and palms placed on the wall. In the sitting position, it is of utmost importance that the upper body is upright at an angle of 90°, and as with the standing position, arms are slightly abducted and hands positioned symmetrically. For assessment in the supine position, it is vital that the patient is lying completely flat (without a pillow under their head) to avoid flexion in the neck and thus obstructing the clavicles and sternal notch.

## 5. Conclusions

The findings of this study support the reliability and correlation of the three-dimensional volumetric assessment of breast surgery patients in the sitting position with the assessment performed on a standing patient. This indicates that intraoperative assessments in the sitting position can be trusted to accurately reflect the actual breast volume, thus providing valuable information for intraoperative adjustments during breast surgery.

On the other hand, the breast volumes measured in the supine position did not statistically correlate with the standing position. This suggests that caution should be exercised when relying upon supine position measurements for intraoperative decision-making and adjustments.

Three-dimensional surface imaging underestimates breast volume in the supine position when compared to validated measurements in the standing position. Considering this, we strongly advise bringing patients to an upright position intraoperatively to correctly assess breast volume and volumetric differences.

## Figures and Tables

**Table 1:** Demographic data for the evaluated patients.

	Patients Mean ± Std (Min, Max)
Age at Surgery	46.5 ± 15.69 (17; 80), *n* = 87
Gender	87 Female
Body Mass Index	24.71 ± 3.96 (18.07; 36), *n* = 87
Diabetes	5/87
Postoperative Breasts	184/200
Preoperative Breasts	16/200

**Table 2:** Mean absolute differences of measured breast volumes per breast between the standing and sitting positions and between the standing and supine positions of the 200 evaluated breasts. Values are expressed as mean ± SD [95% confidence interval].

	Measured Breast Volumes	*p* Value
Mean Absolute Volume Difference Between the Standing and Sitting Positions	7.15 ± 93.67 cc	0.281
	*n* = 200	
Mean Absolute Volume Difference Between the Standing and Supine Positions	120.31 ± 113.06 cc	<0.001
	*n* = 200	

## Data Availability

Supporting data are available from the authors upon request.
